# Impact of partial volume correction on the regional correspondence between in vivo [C-11]PiB PET and postmortem measures of Aβ load

**DOI:** 10.1016/j.nicl.2018.04.007

**Published:** 2018-04-04

**Authors:** Davneet S. Minhas, Julie C. Price, Charles M. Laymon, Carl R. Becker, William E. Klunk, Dana L. Tudorascu, Eric E. Abrahamson, Ronald L. Hamilton, Julia K. Kofler, Chester A. Mathis, Oscar L. Lopez, Milos D. Ikonomovic

**Affiliations:** a Department of Radiology, University of Pittsburgh, Pittsburgh, PA, USA; b Department of Radiology, Massachusetts General Hospital, Boston, MA, USA; c Department of Neurology, University of Pittsburgh, Pittsburgh, PA, USA; d Department of Neuropathology, University of Pittsburgh, Pittsburgh, PA, USA; e Department of Medicine, University of Pittsburgh, Pittsburgh, PA, USA; f Department of Biostatistics, University of Pittsburgh, Pittsburgh, PA, USA; g Department of Psychiatry, University of Pittsburgh, Pittsburgh, PA, USA; h Geriatric Research Education and Clinical Center, Veterans Affairs Pittsburgh Healthcare System, Pittsburgh, PA, USA

**Keywords:** Partial volume correction, Amyloid imaging, PiB, PET

## Abstract

The positron emission tomography (PET) radiotracer Pittsburgh Compound B ([C-11]PiB) demonstrates a high affinity for fibrillary amyloid-beta (Aβ) aggregates. However, [C-11]PiB's in vivo sensitivity and specificity is an ongoing area of investigation in correlation studies with postmortem measures of Aβ pathology. One potential confound in PET-to-postmortem correlation studies is the limited spatial resolution of PET and resulting partial volume effects (PVEs). In this work, we evaluated the impact of three partial volume correction (PVC) techniques – the Meltzer, the modified Müller-Gärtner, and the Region-Based Voxel-Wise – on correlations between region-matched in vivo [C-11]PiB standardized uptake value ratios (SUVRs) and postmortem measures of Aβ pathology in a unique cohort of nine subjects. Postmortem Aβ pathology was assessed histologically as percent area coverage of 6-CN-PiB positive and Aβ immunoreactive (4G8 antibody) deposits. The application of all three PVC techniques resulted in minimally reduced PET-to-postmortem correlations relative to no PVC. However, correlations to both 6-CN-PiB and 4G8 percent area across all PVC techniques and no PVC were statistically significant at *p* < 0.01, suggesting that PVC is of minimal importance in understanding the relationship between Aβ PET and neuropathologically assessed Aβ. Thus, the utility of PVC in Aβ PET imaging should continue to be examined on an application-specific basis.

## Highlights

• Three partial volume correction techniques are applied to PiB PET SUVR data.
• Corrected and uncorrected data are correlated to postmortem measures of Aβ load.
• All 3 correction methods minimally impact PET-to-postmortem correlations.

## 1 Introduction

Alzheimer's disease (AD) is characterized clinically by impaired cognitive function (Förstl and Kurz, 1999) and neuropathologically by extracellular amyloid-beta (Aβ) plaques, intracellular neurofibrillary tangles of hyper-phosphorylated tau protein, and synaptic/neuronal loss resulting in regional hypometabolism and cortical atrophy (Mirra et al., 1991). To facilitate clinical diagnosis and early disease detection, several positron emission tomography (PET) radioligands were developed for imaging Aβ pathology in vivo, including ^11^C-radiolabelled Pittsburgh Compound B ([C-11]PiB) (Klunk et al., 2004; Engler et al., 2002), ^18^F-Florbetapir (Wong et al., 2010), ^18^F-Flutemetamol (Vandenberghe et al., 2010), and ^18^F-florbetaben (Rowe et al., 2008), the latter three of which have been FDA approved for clinical use (FDA approves 18F-florbetapir PET agent, 2012). Although these Aβ PET radioligands have high affinity for fibrillary Aβ aggregates in the grey matter (GM), characterization of their sensitivity and specificity is ongoing.

One important tool in this ongoing characterization is the comparison of in vivo Aβ PET measures with postmortem measures of Aβ deposition commonly held to be the “gold standard” (Bacskai et al., 2007; Ikonomovic et al., 2008; Cairns et al., 2009; Burack et al., 2010; Kadir et al., 2011; Sojkova et al., 2011; Kantarci et al., 2012; Ikonomovic et al., 2012; Driscoll et al., 2012; Seo et al., 2017). These comparisons typically result in good, but imperfect, correlations between the in vivo and postmortem quantifications. This could be due to differences between what is actually detected by the in vivo PET Aβ tracers and the postmortem detection techniques, or it could be due to artifacts introduced by either the in vivo or postmortem analysis methods. Since most postmortem analyses quantify Aβ in microscopic fields limited only to brain cortex, one possible in vivo artifact could be created by the inclusion of tissues outside of the cortex caused by the relatively low spatial resolution of PET. This study looks at the effect of common methods to correct for this unintentional inclusion of non-cortical tissue in PET measurements of Aβ.

The poor spatial resolution of Aβ PET imaging relative to magnetic resonance (MR) imaging and X-ray computed tomography (CT) is due to technical factors including detector size, positron range, and noncollinearity (Saha, 2010). The spatial resolution of PET characterized by a point spread function (PSF) corresponds to the image of a point source and is modeled as a Gaussian function with a defined full width at half maximum (FWHM). Whole-body PET scanner spatial resolutions typically range from 4 mm to 6 mm FWHM. Quantification of radioactivity concentration in structures which are large in comparison to this resolution scale (>2xFWHM) is reasonably accurate (Hoffman et al., 1979). However, in PET brain imaging, volume of interest (VOI) size typically falls below this threshold resulting in reduced measurement accuracy due to the blurring of activity between regions, i.e. activity spill-in/spill-out between adjacent VOIs. Resolution-induced inaccuracy is often referred to as the partial volume effect (PVE) (Hoffman et al., 1979; Mazziotta et al., 1981). PVEs may confound quantification of Aβ PET imaging, particularly in elderly subjects where cortical atrophy, with the expansion of CSF spaces and thinning of cortex, may result in the underestimation of tracer uptake in CSF-bordering cortical grey matter (GM). Furthermore, non-specific white matter (WM) uptake (common to all currently available Aβ PET radioligands) can cross-contaminate cortical GM, potentially inflating the signal in Aβ-free healthy controls or reducing apparent retention signal in AD patients with high Aβ burden.

To address PVEs, several protocols were developed with variable success; these are referred to as partial volume correction (PVC) techniques and include: 1) the Meltzer method, which addresses spill-out of activity from the brain to CSF space but does not account for heterogeneity within tissue (Meltzer et al., 1990; Meltzer et al., 1999; Price et al., 2005; Lopresti et al., 2005); 2) the modified Müller-Gärtner (mMG) method, which addresses cross-contamination between GM and WM but does not account for heterogeneity within WM or GM (Rousset et al., 1998a); 3) the geometric transform matrix (GTM) method (Rousset et al., 1998b); and 4) the Region-Based Voxel-Wise (RBV) method (Thomas et al., 2011). The latter two methods account for within-tissue type heterogeneity through parcellating the brain into contiguous non-overlapping regions. Each of these methods rely on anatomical information typically provided by a co-registered MR image, and model the observed PET image as a convolution of the true image by a point spread function.

The impact of PVC techniques in Aβ PET studies is an ongoing area of investigation. Mikhno et al. (2008) demonstrated that voxel-based analysis with the mMG method improved separation of AD and healthy control groups. Rabinovici et al. (2010) obtained similar results using the Meltzer method in their study of healthy controls, early-onset AD, and late-onset AD. However, Drzezga et al. (2008) observed that when the mMG method was used, differences in [C-11]PiB PET uptake between semantic dementia and AD groups were less prominent. Recently, Su et al. (2015) demonstrated that PVEs, if uncorrected, can lead to underestimated measures of longitudinal change in Aβ pathology in the presence of decreasing cortical thickness. Schwarz et al. (2017) observed that the use of Meltzer PVC increased longitudinal plausibility, that is the percent of subjects not decreasing in [C-11]PiB retention between baseline to follow-up scans. However, another study examining the regional correlations between [C-11]PiB and post-mortem Aβ pathology found correlations were consistent between uncorrected and mMG partial volume-corrected SUVR data (Seo et al., 2017).

In our investigations of the correspondence between [C-11]PiB PET and postmortem measures of Aβ pathology, we previously applied a modified form of the Meltzer PVC method to [C-11]PiB PET measures in two case reports (Ikonomovic et al., 2008; Ikonomovic et al., 2012), but uncorrected [C-11]PiB measures were not examined. In the current work, we compared the effects of three PVC techniques on the correspondence between region-matched in vivo PET and postmortem measures of Aβ pathology in nine subjects who had an in vivo [C-11]PiB PET scan and later underwent postmortem neuropathology examination.

## 2 Materials and methods

### 2.1 Subject data

Nine subjects (*n* = 6 male, *n* = 3 female) with in vivo [C-11]PiB PET and MR scans and postmortem histological assessments of Aβ pathology were included in this study (Table 1). One case has been reported previously (Ikonomovic et al., 2012) (Case#02). Clinical diagnosis of AD was based on a standardized University of Pittsburgh Alzheimer's Disease Research Center (ADRC) evaluation at a Consensus Conference, utilizing Diagnostic and Statistical Manual of Mental Disorders, Fourth Edition (DSM-IV) and National Institute of Neurological and Communicative Disorders and Stroke and the Alzheimer's Disease and Related Disorders Association (NINCDS/ADRDA) criteria (McKhann et al., 1984). Neuropathological diagnosis was determined by a board-certified neuropathologist (RLH or JKK) using Consortium to Establish a Registry for Alzheimer's Disease (CERAD) (Mirra et al., 1991) and National Institute on Aging-Reagan Institute (NIA-RI) consensus (Ronald and G. National Institute, 1998) criteria (Table 1).

**Table 1:** Demographics for nine subjects with postmortem measures of Aβ pathology load and in vivo [C-11]PiB PET and MR scans. Clinical diagnoses include probable Alzheimer's disease (AD), dementia with Lewy bodies (DLB), frontotemporal dementia (FTD), and normal cognition (NC), and were determined through a battery of tests including the Mini Mental State Exam (MMSE).

Subject	Diagnosis	Gender	Age at scan (years)	MMSE at scan	PET-death interval (months)	[C-11]PiB PET scan duration	MR scanner & sequence
Case#01	AD	Male	58	18	42.3	0–90 min	GE Signa 1.5 T SPGR
Case#02	DLB	Male	77	10	17.2	0–90 min	GE Signa 1.5 T SPGR
Case#03	AD	Male	54	19	30.4	0–90 min	GE Signa 1.5 T SPGR
Case#04	AD	Male	74	21	10.5	0–90 min	GE Signa 1.5 T SPGR
Case#05	AD	Female	66	21	34.6	0–90 min	GE Signa 1.5 T SPGR
Case#06	FTD	Male	80	7	37.2	0–90 min	GE Signa 1.5 T SPGR
Case#07	AD	Female	79	25	45.5	0–90 min	GE Signa 1.5 T SPGR
Case#08	NC	Female	80	28	31.8	40–70 min	GE Signa 1.5 T SPGR
Case#09	NC	Male	85	29	37.4	40–70 min	Siemens Tim Trio 3 T MPRG

Based on the last in vivo clinical diagnosis at time of scan, five subjects had probable AD, one subject had dementia with Lewy Bodies (DLB), one subject had frontotemporal dementia (FTD), and two subjects had normal cognition (NC). At the time of [C-11]PiB PET scan, Mini-Mental State Examination (MMSE) scores in the study cohort ranged from 29 to 7 and age ranged from 54 years to 85 years. The mean interval from time of scan to death was 31.9 ± 11.4 months (see Table 1).

### 2.2 [C-11]PiB PET and MR imaging

All subjects underwent [C-11]PiB PET and MR imaging prior to death (see Table 1 for imaging details). Subjects received a spoiled gradient recalled MR scan (1.5 T, GE Signa) (*n* = 8) or a magnetization prepared rapid gradient echo MR scan (3 T, Siemens Tim Trio) (*n* = 1) for anatomic VOI definition and PVC tissue segmentation guidance. PET data were acquired as previously described (Price et al., 2005; Lopresti et al., 2005) using a Siemens/CTI ECAT HR+ scanner (3-dimensional mode, 63 image planes, 15.2 cm field of view) following slow bolus injection of 14.9 ± 1.7 kBq of high specific activity (>21.4 GBq/μmol) [C-11]PiB. PET emission data were acquired over 0–90 min (34 frames, *n* = 7) or 40–70 min (6 frames, *n* = 2) post injection. PET data were corrected for attenuation, scatter, and radioactivity decay, and reconstructed using the Direct Fourier (DIFT) method, similar to filtered backprojection, with a 3 mm Hann filter into a 128 × 128 × 63 matrix with voxel sizes of 2.06 × 2.06 × 2.43 mm^3^. The reconstructed PET image resolution was approximately 6 mm FWHM in the transverse and axial planes, measured using a point source phantom imaged in the center of the PET scanner field of view (FOV).

### 2.3 Brain autopsy and dissection

This study was approved by the University of Pittsburgh Institutional Review Board and the University of Pittsburgh's Committee for Oversight of Research and Clinical Training Involving Decedents. Written informed consent for research and autopsy was obtained for all subjects in the study. Brain autopsies were performed under a University of Pittsburgh Alzheimer's Disease Research Center protocol, as previously described (Ikonomovic et al., 2008; Ikonomovic et al., 2012). For each case, the left cerebral hemisphere was immersed in 10% buffered formalin for 21 days, and then sliced into 1-cm thick axial blocks. CERAD designated brain regions were sampled for diagnostic purposes.

To quantify regional plaque load in the precuneus, tissue sections were dissected from 10% formalin-fixed axial tissue blocks and processed for histology and immunohistochemistry as described below.

### 2.4 Histology and immunohistochemistry

Histofluorescent labeling with 6-CN-PiB (10 μM), a highly fluorescent derivative of [C-11]PiB (Ikonomovic et al., 2008; Mathis et al., 2003), and immunohistochemistry with 4G8, an antibody recognizing aa17-24 of Aβ (Ikonomovic et al., 2012), in the precuneus were performed using previously published protocols (Ikonomovic et al., 2008). Aβ pathology load (percent area of GM occupied by 6-CN-PiB-labeled or 4G8-immunoreactive Aβ deposits) was quantified using NIH Image (Rasband, W.S., ImageJ, US National Institutes of Health, Bethesda, MD, USA, http://rsb.info.nih.gov/ij/, 1997–2017) on three pairs of 40 μm-thick adjacent tissue sections equally spaced through the VOI tissue block as previously described (Ikonomovic et al., 2012). Additional adjacent tissue sections were processed using cresyl violet to delineate the boundary between GM and WM.

### 2.5 Volume of interest (VOI) matching in PET and postmortem tissues

**Fig. 1:**
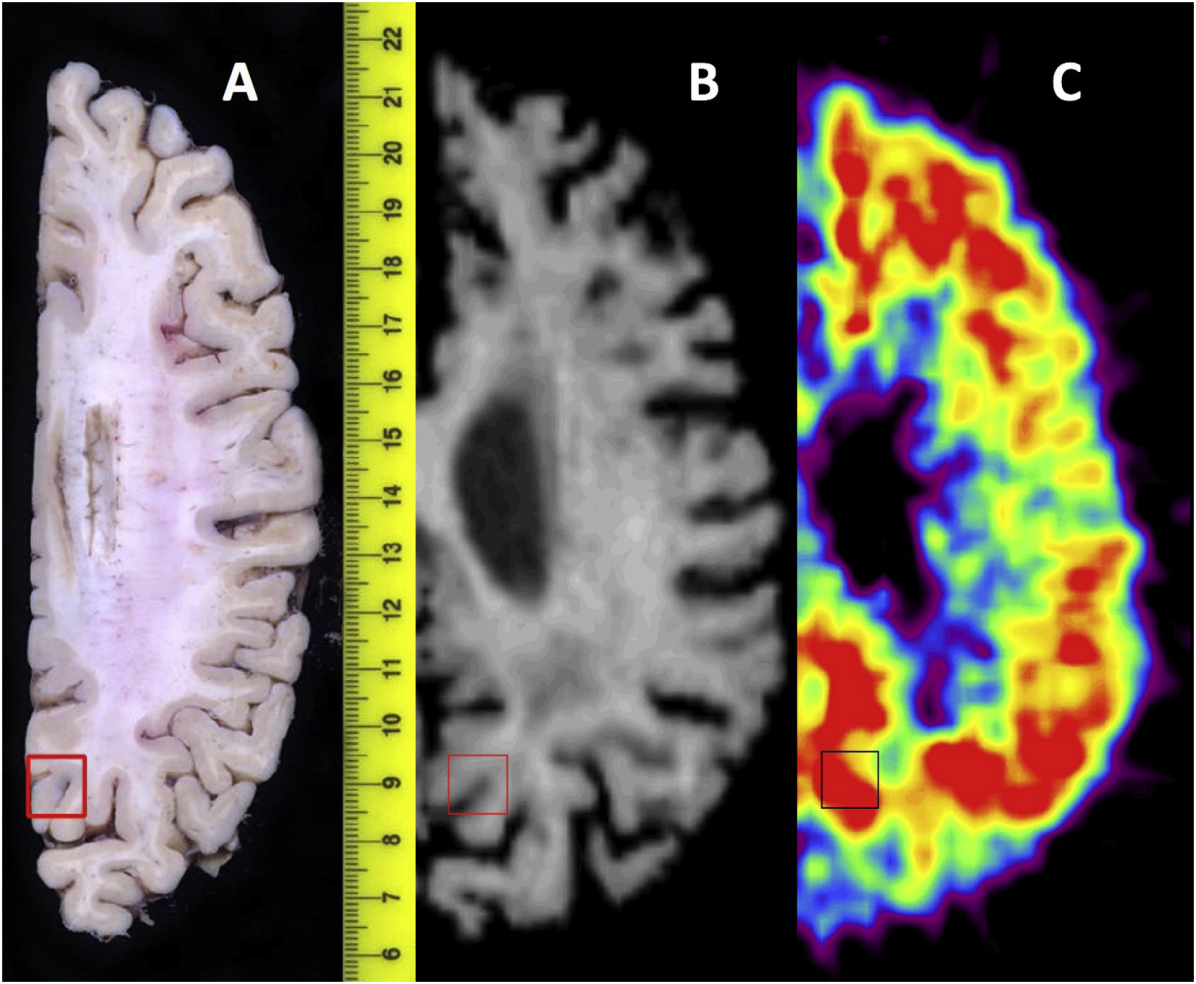
Snapshot of precuneus VOI matching between post-mortem axial tissue block and matching in vivo MR image for Case#07. From left-to-right, (A) is the post-mortem autopsy tissue block with excised region boxed, (B) is the in vivo MR image with VOI hand-drawn to match that in the autopsy photo, and (C) is the [C-11]PiB SUVR image with the hand-drawn VOI overlaid.

Matching of the autopsy precuneus VOI tissue block to the VOI sampling of the dynamic PET image data in the same region was based on a previously described method (Ikonomovic et al., 2008). VOI-labeled photographs of axial autopsy blocks guided VOI generation on the in vivo full-resolution axial MR image. Each subject's MR image was manually reoriented to match the orientation of the autopsy slice photograph, and VOIs were generated on these reoriented MR images to match those on the autopsy tissue. Adjustments to VOI placement on MR images were necessary due to postmortem collapsing of the ventricles after drainage of cerebral spinal fluid (CSF) and due to tissue deformation during fixation (see Fig. 1).

Frame-to-frame motion within dynamic PET images was visually assessed by applying a contour to a mid-time point frame delineating the pial surface and ventricles of the brain in ROITool software (CTI PET Systems Knoxville, TN, USA). If interframe motion was observed, a set of stable frames was averaged and used as reference for frame-to-frame registration using automated techniques previously described (Price et al., 2005). After motion assessment, each subject's dynamic PET image was coregistered to the reoriented MR image using automated methods, and VOI maps were transferred from MR image to PET (Price et al., 2005). A standardized uptake value (SUV) 50–70 min parametric image was then generated from the co-registered PET by averaging activity over the appropriate time frames on a voxel basis, then multiplying by the subject's weight and dividing by injected dose.

### 2.6 Partial volume correction application and sampling

**Fig. 2:**
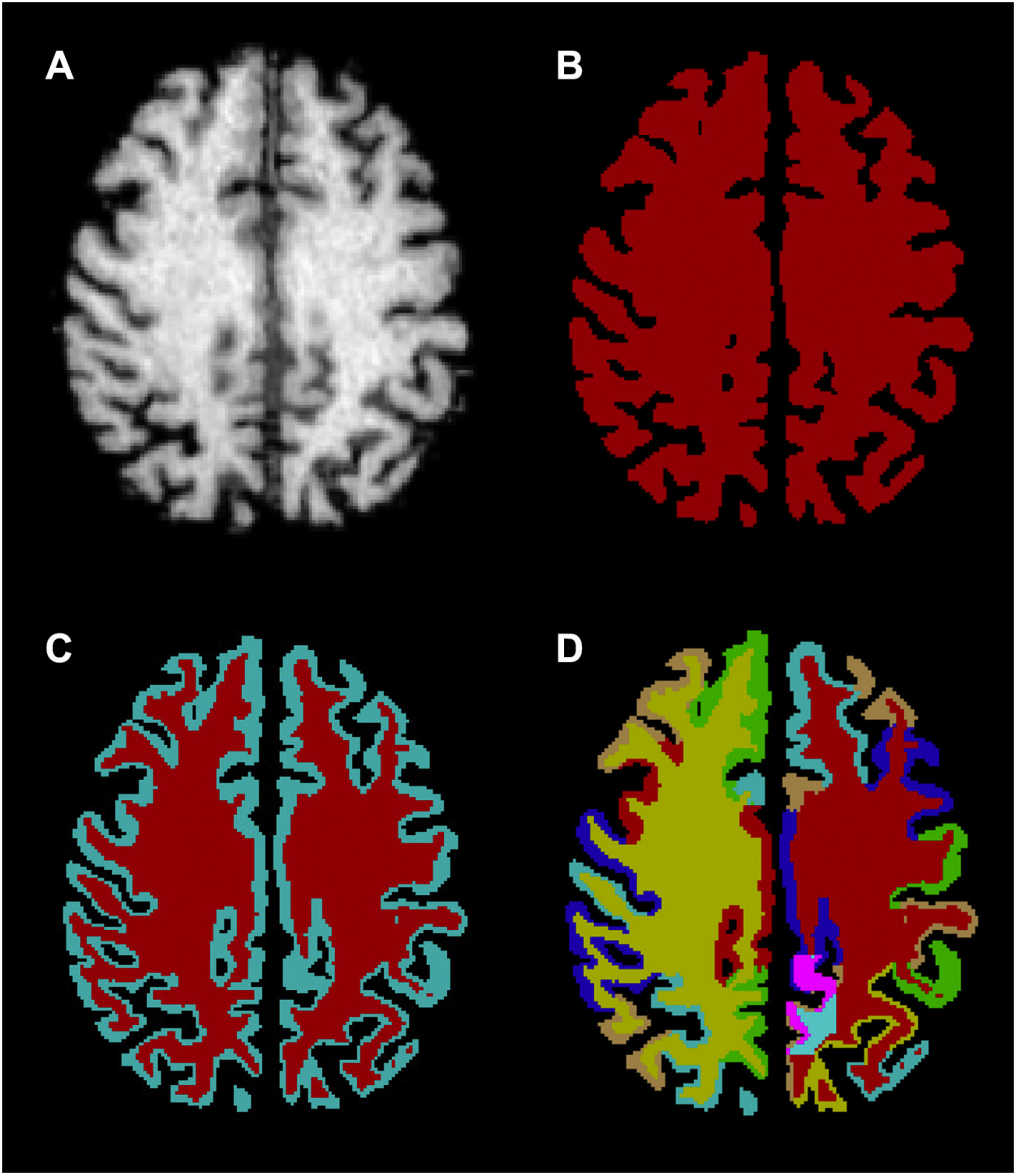
Case#01 MR scan and FreeSurfer segmentations and parcellations. (A) Case#01 MR scan. (B) Case#01 tissue binary mask (red) for use in Meltzer PVC. (C) Case#01 GM binary mask (light blue) and WM binary mask (red) for use in mMG PVC. (D) Case#01 total FreeSurfer parcellation with the hand-drawn precuneus GM VOI (pink) inserted for use in RBV PVC. (For interpretation of the references to color in this figure legend, the reader is referred to the web version of this article.)

Each subject's reoriented MR image was parcellated using FreeSurfer v5.3 (Fischl et al., 2002; Fischl et al., 2004). Each FreeSurfer parcellation was visually inspected and manually edited to ensure GM, WM, CSF, and within-tissue anatomical boundaries were followed. Parcellations were combined into 86 anatomical regions and converted to binary masks. These 86 contiguous, non-overlapping binary masks spanned the entire brain and consisted of lateralized cortical and subcortical GM regions, cerebral WM, cerebellar GM, cerebellar WM, and brainstem. The binary masks were also combined to create full GM and WM tissue masks, both of which in turn were combined to create a single whole-brain tissue mask (Fig. 2B, C).

For each subject, a voxel-level Meltzer-corrected [C-11]PiB SUV image was generated from the subject's [C-11]PiB SUV parametric image and the whole-brain tissue mask, as described by Meltzer et al. (1990). A voxel-level mMG-corrected SUV image was generated from the SUV parametric image and the GM and WM tissue masks (Rousset et al., 1998a).

The autopsy-based precuneus VOIs were also incorporated into the contiguous, non-overlapping 86 binary masks. Any subregion generated by the intersection of an autopsy-based VOI and binary mask was excluded from the binary mask and treated as a new, separate binary mask. This FreeSurfer- and autopsy-derived binary mask parcellation of the brain (Fig. 2D) was used to generate a voxel-level RBV-corrected [C-11]PiB SUV image (Thomas et al., 2011).

Each subject's autopsy-based VOIs were segmented into GM and WM portions using the FreeSurfer-derived GM and WM binary masks. The four SUV parametric images for each subject (not PV-corrected, Meltzer-corrected, mMG-corrected, and RBV-corrected) were then sampled using GM-only VOIs to mimic the Aβ pathology load analysis, which was assessed exclusively in the GM of each tissue block. For each subject, standardized uptake value ratios (SUVR) were produced by normalizing the VOI SUV to the equivalently corrected or uncorrected SUV value of the FreeSurfer-delineated cerebellar GM.

### 2.7 Statistical analyses

Precuneus regional correlation between in vivo [C-11]PiB SUVR and both postmortem 6-CN-PiB positive Aβ pathology load (percent area) and 4G8-immunolabeled Aβ pathology load (percent area) across all subjects was evaluated using Pearson's correlation coefficient. Statistical significance was set at *p* < 0.01 (two-sided).

## 3 Results

### 3.1 [C-11]PiB PET outcome measures

**Fig. 3:**
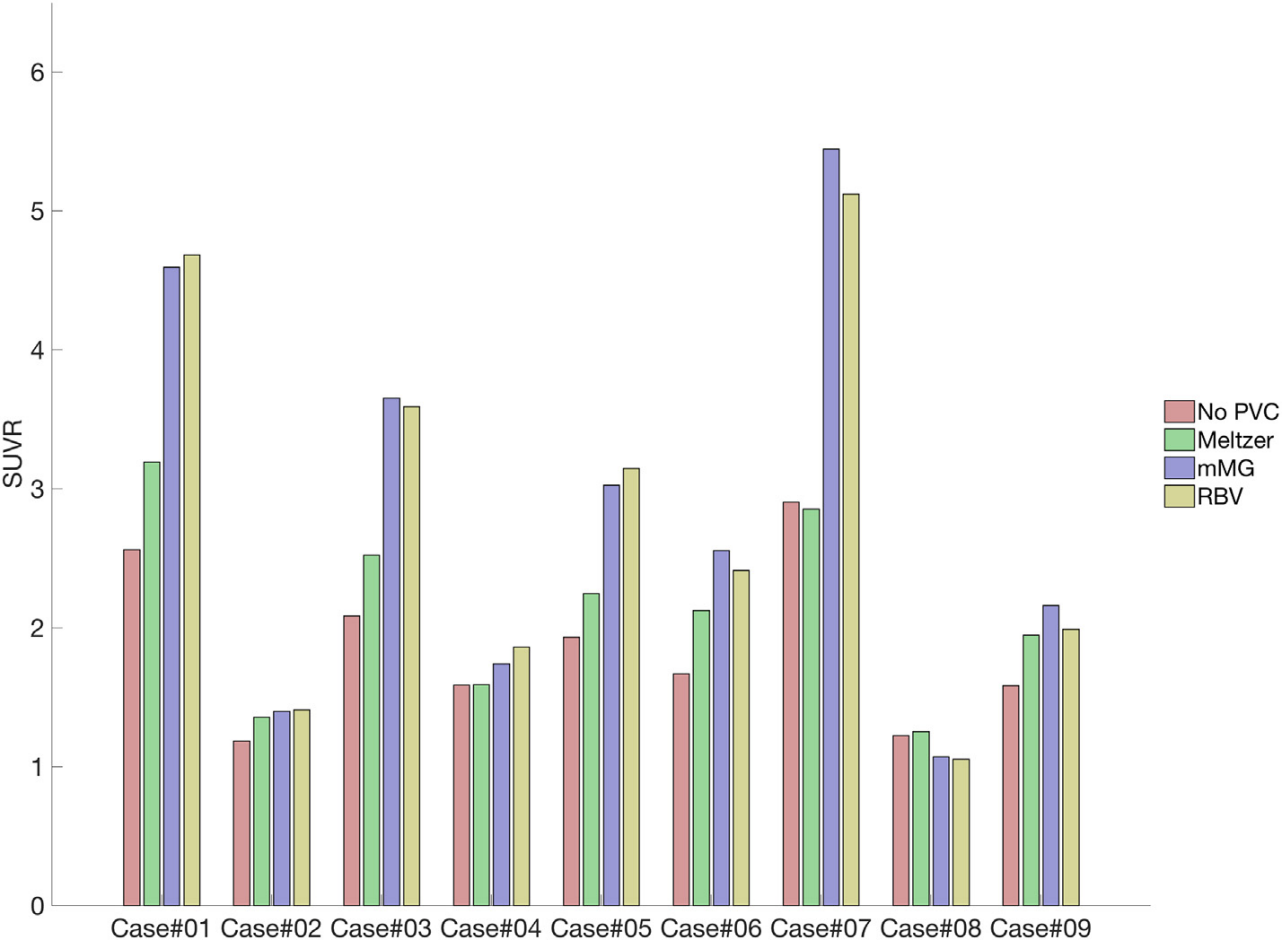
Mean precuneus SUVR values across subjects and partial volume correction methods. On average, the Meltzer PVC method increased precuneus SUVR values by 14.6%. The mMG and RBV methods increased SUVR values on average by 46.2% and 44.3%, respectively.

Fig. 3 shows mean SUVR values for the precuneus region across all subjects and the three PVC techniques. On average, the Meltzer PVC method increased precuneus SUVR values by 14.2%. SUVR values were increased to a greater extent using the mMG method (44.8%) and the RBV PVC method (42.9%). PVC increased measured SUVR in all subjects, with the exception of Case#07, which had small reductions in SUVR (1.7%) after correction with the Meltzer method, and Case#08, where SUVR reductions ranged from 12.3%–13.9% for both mMG and RBV methods. The largest increases in SUVR due to PVC also occurred in Case#07 (87.4% increase from mMG PVC) and Case#01 (82.6% increase from RBV PVC).

### 3.2 Correlations between in vivo [C-11]PiB PET and postmortem Aβ load

**Fig. 4:**
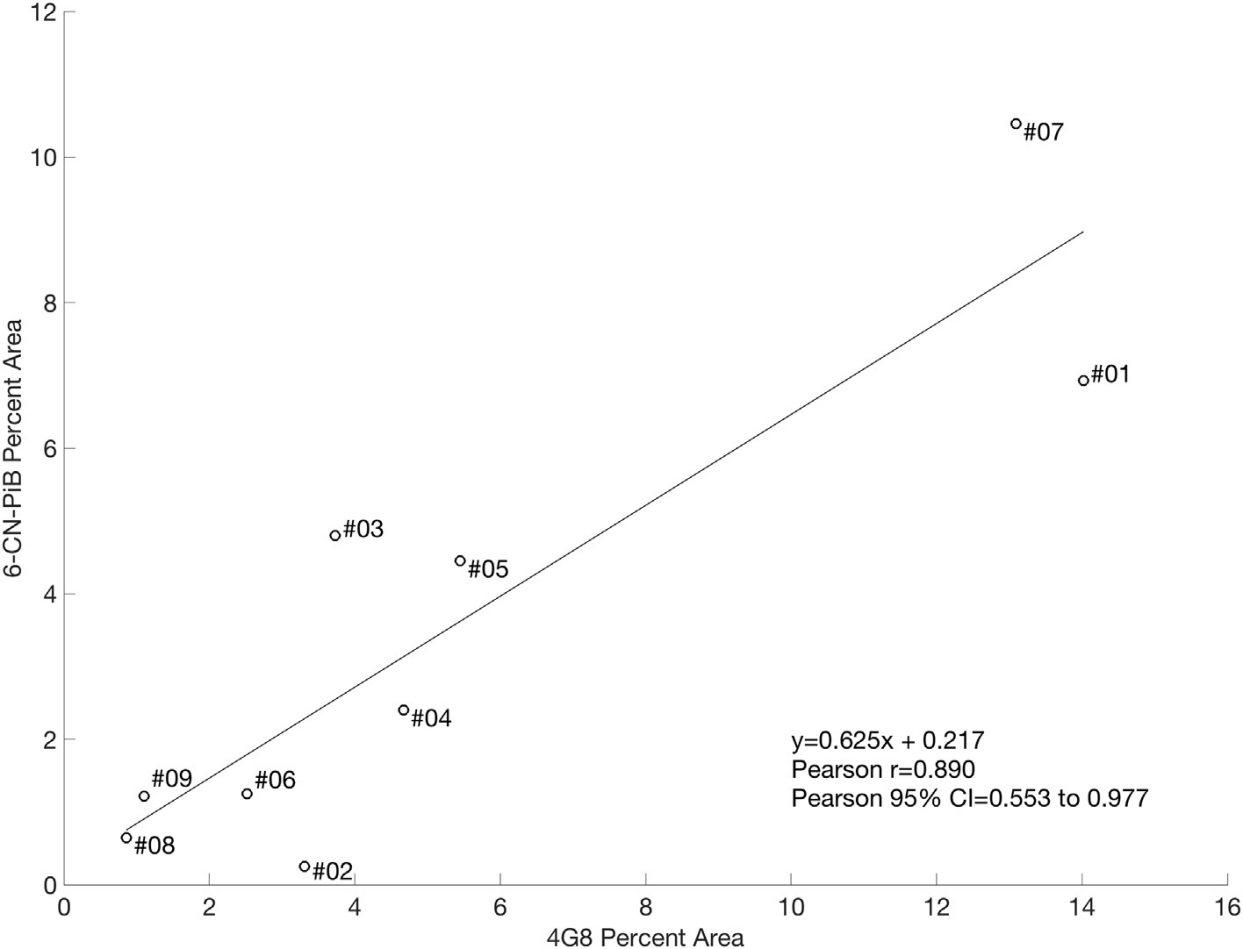
Correlation between precuneus postmortem Aβ pathology measures using 4G8 percent area and 6-CN-PiB percent area. 4G8 and 6-CN-PiB are well correlated in this sample (Pearson *r* = 0.89, *p* < 0.01).

Postmortem measures of Aβ pathology load (percent area) using 6-CN-PiB and 4G8 across all subjects in the study were highly correlated at *p* < 0.01 (Fig. 4).

**Fig. 5:**
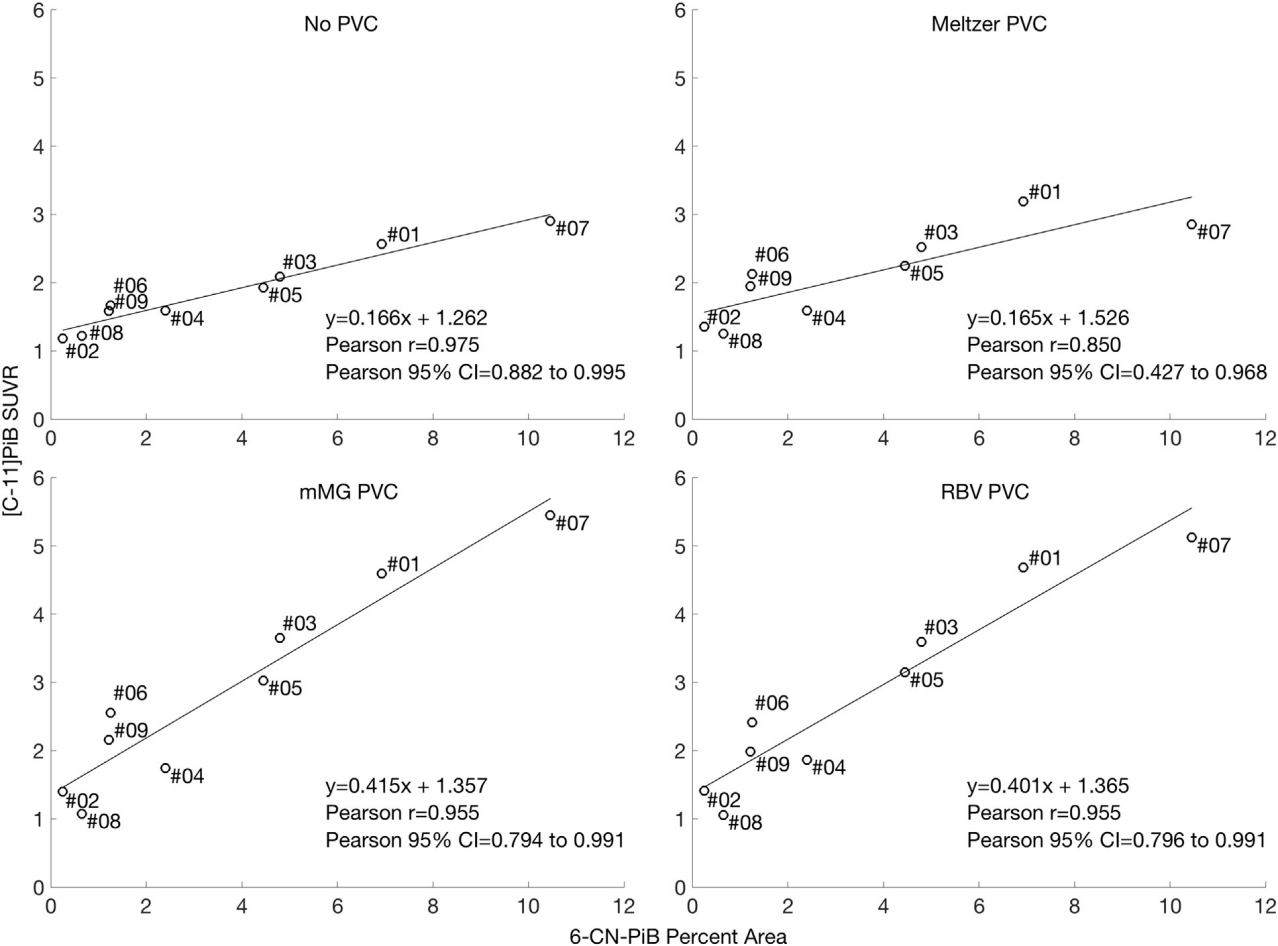
Correlations between precuneus [C-11]PiB SUVR and 6-CN-PiB positive Aβ pathology (percent area) across subjects. Each point is labeled with the subject's case number. All correlations are statistically significant at *p* < 0.01.

Correlations between postmortem 6-CN-PiB Aβ pathology load (percent area) and in vivo SUVR measures are shown in Fig. 5. All partial volume correction methods minimally reduced the correlation between 6-CN-PiB and SUVR, but all correlations remain statistically significant at *p* < 0.01. 6-CN-PiB measures for the precuneus ranged from 0.25% area (Case#02, DLB) to 10.46% area (Case#07, AD).

**Fig. 6:**
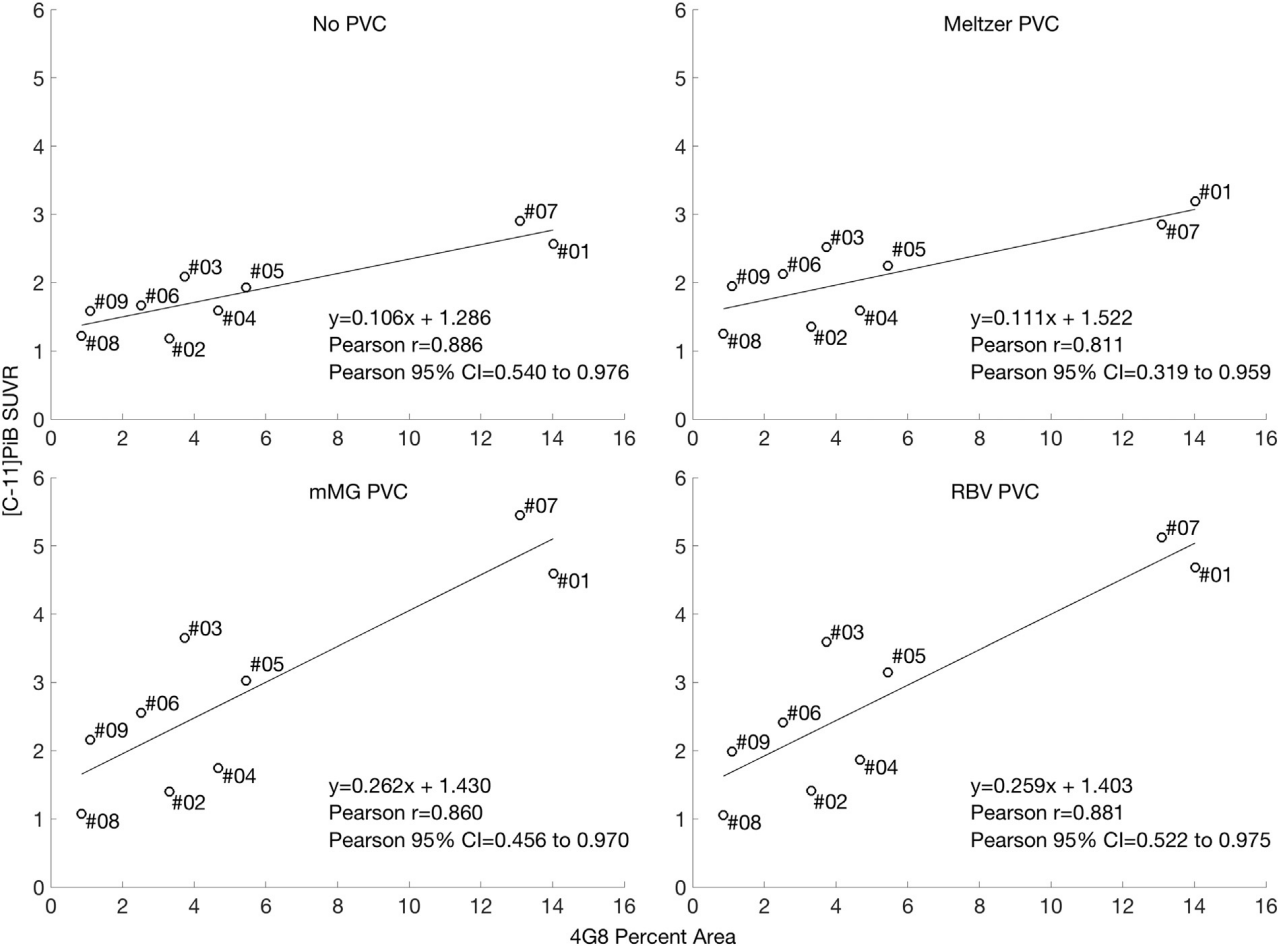
Correlations between precuneus [C-11]PiB SUVR and 4G8-immunoreactive Aβ pathology (percent area) across subjects. Each point is labeled with the subject's case number. All correlations are statistically significant at *p* < 0.01.

Correlations between postmortem 4G8-immunoreactive Aβ pathology load (percent area) and in vivo [C-11]PiB SUVR for the precuneus were similarly ranked across PVC techniques, with no PVC resulting in the highest correlation to 4G8-immunoreactive Aβ pathology load (Fig. 6). Again however, all correlations were statistically significant at *p* < 0.01. 4G8 measures ranged from 0.86% area (Case#08) to 14.02% area (Case#01).

## 4 Discussion

In this work, we examined the impact of three PVC techniques on [C-11]PiB PET measures and the correlation of [C-11]PiB PET measures with postmortem Aβ pathology load.

Averaged over subjects, the use of the Meltzer PVC technique resulted in approximately a 14% increase in precuneus SUVR values, consistent with a previously reported ~22% increased average [C-11]PiB uptake using the same method in a study of late-onset AD (Rabinovici et al., 2010). Both the mMG and RBV correction methods in this work resulted in 44.8% to 42.9% increased precuneus SUVR values, similar to 44% (mMG corrected) and 56% (RBV corrected) increases observed in a previous study of AD subjects (Thomas et al., 2011). Although previous studies have generally reported increases in [C-11]PiB retention measures after PVC (Thomas et al., 2011; Mikhno et al., 2008; Rabinovici et al., 2010; Su et al., 2015), we observed reduced precuneus [C-11]PiB SUVR in two subjects. Application of the Meltzer method resulted in small decreases in precuneus SUVR in Case#07 (AD), while the use of both mMG and RBV correction methods resulted in relatively large reductions in precuneus SUVR in Case#08 (NC). In the first case, the Meltzer method had a larger impact on the cerebellar reference region than the precuneus SUV measure, increasing cerebellar GM SUV by 25%, slightly higher than the 22–23% increases in the precuneus SUV. A similar phenomenon was reported by Su et al. (2015) (Su et al., 2015), who observed that the Meltzer correction method resulted in increased cerebellar cortex intensities but lower putamen binding potential values in specific subjects. In the current study, substantial cerebellar atrophy was apparent in the MR image for Case#07, which likely led to the larger increase in cerebellar SUV and therefore lower precuneus SUVR.

The reductions in precuneus SUVR values for Case#08 after both mMG- and RBV-correction are likely due to this case being a normal control subject with low specific binding of [C-11]PiB in GM. Non-specific signal from WM was relatively greater and likely spilled over into the precuneus GM voxels. Both the mMG and RBV methods correct for the partial volume effect from WM, so activity assumed to originate from WM is removed from GM voxels in the VOI, resulting in lower GM activity.

Collectively, these cases highlight the fact that the impact of any PVC technique is highly region- and subject-specific. MR image segmentation or parcellation, PET-to-MR image coregistration, and VOI placement must all be performed consistently and cautiously when applying any PVC technique in Aβ PET imaging, given the differing levels of atrophy and variable GM-to-WM binding ratios.

Despite having varied effects on [C-11]PiB PET SUVR in this small and diverse sample of subjects, the PVC techniques examined ranked similarly in their impact on correlations to 6-CN-PiB and to 4G8 measures of Aβ pathology load. This is expected given the strong correlation between 6-CN-PiB and 4G8 percent area coverage. Each PVC method resulted in minimally reduced correlations relative to no PVC, with RBV correction being the greatest, followed by mMG and Meltzer. Nevertheless, PET-to-postmortem correlations across all PVC methods and no PVC remained highly statistically significant at *p* < 0.01. These results agree with Seo et al. (2017), who also found that Müller-Gärtner correction had minimal impact on correlations between [C-11]PiB PET and postmortem measures of Aβ pathology load (Seo et al., 2017), and suggest the utility of PVC in Aβ PET imaging is application-specific and should continue to be examined. While previous studies have found PVC may be advantageous in discriminating diagnostic groups (Mikhno et al., 2008; Rabinovici et al., 2010) or tracking longitudinal change (Su et al., 2015; Schwarz et al., 2017), our results suggest PVC is of minimal importance in understanding the relationship between Aβ PET and Aβ pathology.

One limitation of this study is the small sample size. Additional subjects could significantly change PET-to-postmortem correlations, and the impact of PVC on correlations should be examined in a larger cohort. The inconsistent time intervals between PET scan and autopsy in this population is also a potential source of variability, as Aβ pathology may have accumulated beyond a subject's last [C-11]PiB PET scan, particularly in the clinically mild-moderate AD subjects examined in this study. Inaccuracies in tissue matching between postmortem excisions and VOIs on MR images, a particularly difficult task given the deformations brains undergo at autopsy as evidenced in Fig. 1, could contribute further variability. Finally, each of the PVC methods applied here are also susceptible to errors in PET-to-MR image registration and segmentation (Meltzer et al., 1999; Frouin et al., 2002). Despite these limitations and potential sources of error, correlations between [C-11]PiB PET SUVR and both 6-CN-PiB percent area and 4G8 percent area were highly statistically significant with and without partial volume correction in this study.

## 5 Conclusions

The Meltzer, modified Müller-Gärtner, and Region-Based Voxel-Wise partial volume correction techniques each had minimal impact on the correlation between [C-11]PiB PET SUVR and postmortem measures of Aβ pathology load, resulting in statistically insignificant reductions in PET-to-postmortem correlations. Thus, the utility of PVC should continue to be explored and questioned on an application-specific basis.

## References

[bb0005] Bacskai B.J. (2007). Molecular imaging with Pittsburgh compound B confirmed at autopsy: a case report. Arch. Neurol..

[bb0010] Burack M.A. (2010). In vivo amyloid imaging in autopsy-confirmed Parkinson disease with dementia. Neurology.

[bb0015] Cairns N.J. (2009). Absence of Pittsburgh compound B detection of cerebral amyloid {beta} in a patient with clinical, cognitive, and cerebrospinal fluid markers of Alzheimer disease: a case report. Arch. Neurol..

[bb0020] Driscoll I. (2012). Correspondence between in vivo (11)C-PiB-PET amyloid imaging and postmortem, region-matched assessment of plaques. Acta Neuropathol..

[bb0025] Drzezga A. (2008). Imaging of amyloid plaques and cerebral glucose metabolism in semantic dementia and Alzheimer's disease. NeuroImage.

[bb0030] Engler H. (2002). First human study with a benzothiazole amyloid-imaging agent in Alzheimer's disease and control subjects. Neurobiol. Aging.

[bb0035] Fischl B. (2002). Whole brain segmentation: automated labeling of neuroanatomical structures in the human brain. Neuron.

[bb0040] Fischl B. (2004). Automatically parcellating the human cerebral cortex. Cereb. Cortex.

[bb0045] Förstl H., Kurz A. (1999). Clinical features of Alzheimer's disease. Eur. Arch. Psychiatry Clin. Neurosci..

[bb0050] Frouin V. (2002). Correction of partial-volume effect for PET striatal imaging: fast implementation and study of robustness. J. Nucl. Med..

[bb0055] Hoffman E.J., Huang S.-C., Phelps M.E. (1979). Quantitation in positron emission computed tomography: 1. Effect of object size. J. Comput. Assist. Tomogr..

[bb0060] Ikonomovic M.D. (2008). Post-mortem correlates of in vivo PiB-PET amyloid imaging in a typical case of Alzheimer's disease. Brain.

[bb0065] Ikonomovic M.D. (2012). Early AD pathology in a [C-11] PiB-negative case: a PiB-amyloid imaging, biochemical, and immunohistochemical study. Acta Neuropathol..

[bb0070] (2012). J. Nucl. Med..

[bb0075] Kadir A. (2011). Positron emission tomography imaging and clinical progression in relation to molecular pathology in the first Pittsburgh compound B positron emission tomography patient with Alzheimer's disease. Brain.

[bb0080] Kantarci K. (2012). Ante mortem amyloid imaging and β-amyloid pathology in a case with dementia with Lewy bodies. Neurobiol. Aging.

[bb0085] Klunk W.E. (2004). Imaging brain amyloid in Alzheimer's disease with Pittsburgh compound-B. Ann. Neurol..

[bb0090] Lopresti B.J. (2005). Simplified quantification of Pittsburgh compound B amyloid imaging PET studies: a comparative analysis. J. Nucl. Med..

[bb0095] Mathis C.A. (2003). Synthesis and evaluation of 11C-labeled 6-substituted 2-arylbenzothiazoles as amyloid imaging agents. J. Med. Chem..

[bb0100] Mazziotta J.C. (1981). Quantitation in positron emission computed tomography: 5. Physical-anatomical effects. J. Comput. Assist. Tomogr..

[bb0105] McKhann G. (1984). Clinical diagnosis of Alzheimer's disease: report of health and human services task force on Alzheimer's disease. Neurology.

[bb0110] Meltzer C.C. (1990). Correction of PET data for partial volume effects in human cerebral cortex by MR imaging. J. Comput. Assist. Tomogr..

[bb0115] Meltzer C.C. (1999). Comparative evaluation of MR-based partial-volume correction schemes for PET. J. Nucl. Med..

[bb0120] Mikhno A. (2008). Voxel-based analysis of 11C-PIB scans for diagnosing Alzheimer's disease. J. Nucl. Med..

[bb0125] Mirra S.S. (1991). The Consortium to Establish a Registry for Alzheimer's Disease (CERAD) part II. Standardization of the neuropathologic assessment of Alzheimer's disease. Neurology.

[bb0130] Price J.C. (2005). Kinetic modeling of amyloid binding in humans using PET imaging and Pittsburgh compound-B. J. Cereb. Blood Flow Metab..

[bb0135] Rabinovici G.D. (2010). Increased metabolic vulnerability in early-onset Alzheimer's disease is not related to amyloid burden. Brain.

[bb0140] Ronald T., G. National Institute on Aging Working (1998). Consensus report of the working group on: “Molecular and biochemical markers of Alzheimer's disease”. Neurobiol. Aging.

[bb0145] Rousset O., Carson R.E., Daube-Witherspoon M.E., Herscovitch P. (1998). Pixel-versus region-based partial volume correction in PET. Quantitative Functional Imaging With Positron Emission Tomography.

[bb0150] Rousset O.G., Ma Y., Evans A.C. (1998). Correction for partial volume effects in PET: principle and validation. J. Nucl. Med..

[bb0155] Rowe C.C. (2008). Imaging of amyloid β in Alzheimer's disease with 18F-BAY94-9172, a novel PET tracer: proof of mechanism. Lancet Neurol.

[bb0160] Saha G.B. (2010). Performance characteristics of PET scanners. Basics of PET Imaging.

[bb0165] Schwarz C.G. (2017). Optimizing PiB-PET SUVR change-over-time measurement by a large-scale analysis of longitudinal reliability, plausibility, separability, and correlation with MMSE. NeuroImage.

[bb0170] Seo S.W. (2017). Regional correlations between [11 C] PIB PET and post-mortem burden of amyloid-beta pathology in a diverse neuropathological cohort. NeuroImage Clin..

[bb0175] Sojkova J. (2011). In vivo fibrillar beta-amyloid detected using C-11 PiB positron emission tomography and neuropathologic assessment in older adults. Arch. Neurol..

[bb0180] Su Y. (2015). Partial volume correction in quantitative amyloid imaging. NeuroImage.

[bb0185] Thomas B.A. (2011). The importance of appropriate partial volume correction for PET quantification in Alzheimer's disease. Eur. J. Nucl. Med. Mol. Imaging.

[bb0190] Vandenberghe R. (2010). 18F-flutemetamol amyloid imaging in Alzheimer disease and mild cognitive impairment: a phase 2 trial. Ann. Neurol..

[bb0195] Wong D.F. (2010). In vivo imaging of amyloid deposition in Alzheimer disease using the radioligand 18F-AV-45 (flobetapir F 18). J. Nucl. Med..

